# Complete Genome Sequence of *Psychrobacter alimentarius* PAMC 27889, a Psychrophile Isolated from an Antarctic Rock Sample

**DOI:** 10.1128/genomeA.00704-16

**Published:** 2016-07-21

**Authors:** Jaejin Lee, Miye Kwon, Jae Young Yang, Jusun Woo, Hong Kum Lee, Soon Gyu Hong, Ok-Sun Kim

**Affiliations:** a Division of Polar Life Sciences, Korea Polar Research Institute, Incheon, Republic of Korea; b Division of Polar Earth-System Sciences, Korea Polar Research Institute, Incheon, Republic of Korea

## Abstract

*Psychrobacter alimentarius* PAMC 27889, a Gram-negative, psychrophilic bacterium, was isolated from an Antarctic rock sample. Here, we report the complete genome of *P. alimentarius* PAMC 27889, which has the nonmevalonate methylerythritol phosphate pathway of terpenoid biosynthesis and a complete gene cluster for benzoate degradation.

## GENOME ANNOUNCEMENT

*Psychrobacter alimentarius*, a Gram-negative, nonmotile, non-spore-forming, moderately halophilic bacterium, was first isolated from fermented seafood (1). Members of the genus *Psychrobacter* have been found in various habitats, such as food, poultry, fish, clinical sources, seawater, and cold environments (i.e., Antarctic soil and Arctic marine environments) (2). Several *Psychrobacter* strains are known to produce industrially useful enzymes, including proteases, esterases, lipases, and dehydrogenases (3, 4). Particularly, cold-adapted enzymes from psychrophilic *Psychrobacter* may have commercial potential because of their high catalytic activity and low energy consumption at low temperatures (5). Here, we report the genome sequence of *P. alimentarius* PAMC 27889, which was isolated from a rock sample collected at Eureka Spurs in Northern Victoria Land, Antarctica (72°41′50″ S, 165°59′40″ E).

Genomic DNA was extracted from *P. alimentarius* PAMC 27889 using the i-genomic BYF minikit (iNtRON Biotechnology, Republic of Korea), and a standard PacBio library with 20-kb average inserts was prepared. Genome sequencing was conducted using Pacific Biosciences (PacBio) RS II single-molecule real-time (SMRT) sequencing technology (Pacific Biosciences, USA) (6). The hierarchical genome assembly process (HGAP) pipeline in the SMRT analysis software version 2.3.0 (7) was used for *de novo* assembly of 67,890 reads averaging 10,062 nucleotides (683,119,892 bp in total), which resulted in one circular chromosome (185-fold coverage) and one circular plasmid (119-fold coverage). The prediction of protein-coding sequences (CDSs) was performed with Prodigal version 2.6.1 (8). The predicted genes were functionally annotated using the UniProt (9), Pfam (10), and COG (11) databases. The genome of *P. alimentarius* PAMC 27889 consists of one circular chromosome of 3,332,539 bp and one plasmid (designated pP27889) of 16,905 bp with 42.9% and 39.4% G+C content, respectively. Those include 2,677 CDSs (i.e., 2,119 of the CDSs were assigned to a putative function and the remaining were annotated as hypothetical proteins), 50 tRNAs, and 15 rRNAs.

According to the annotation results, the genome of *P. alimentarius* PAMC 27889 encodes enzymes involved in the nonmevalonate methylerythritol phosphate pathway of terpenoid biosynthesis. Through the pathway, geranyl pyrophosphate, which is the key precursor of terpenoid molecules, is produced from d-glyceraldehyde 3-phosphate and pyruvate using 1-deoxy-d-xylulose-5-phosphate synthase, 1-deoxy-d-xylulose-5-phosphate reductoisomerase, 2-*C*-methy-d-erythritol 4-phosphate cytidylyltransferase, 4-diphosphocytidyl-2-*C-*methyl-d-erythritol kinase, 2-*C*-methyl-d-erythritol 2,4-cyclodiphosphate synthase, 4-hydroxy-3-methybut-2-en-1-yl diphosphate synthase, 4-hydroxy-3-methylbut-2-enyl diphosphate reductase, and geranylgeranyl diphosphate synthase. A complete gene cluster for benzoate degradation, which degrades benzoate to catechol and subsequently to acetyl-CoA, was also present in the genome. The plasmid, pP27889, possesses l-lactate dehydrogenase and alcohol dehydrogenase for pyruvate fermentation.

### Nucleotide sequence accession numbers.

The genome sequence of *P. alimentarius* PAMC 27889 has been deposited at GenBank under the accession numbers CP014945 (chromosome) and CP014946 (plasmid). The strain PAMC 27889 is available from the Polar and Alpine Microbial Collection (Korea Polar Research Institute, Incheon, Republic of Korea).
